# Effective dose in medicine

**DOI:** 10.1177/0146645320927849

**Published:** 2020-11-04

**Authors:** C.J. Martin

**Affiliations:** Department of Clinical Physics and Bioengineering, University of Glasgow, Gartnavel Royal Hospital, Glasgow G12 0XH, UK

**Keywords:** Effective dose, Risk calculation, Patient dose

## Abstract

The International Commission on Radiological Protection (ICRP) developed effective dose as a quantity related to risk for occupational and public exposure. There was a need for a similar dose quantity linked to risk for making everyday decisions relating to medical procedures. Coefficients were developed to enable the calculation of doses to organs and tissues, and effective doses for procedures in nuclear medicine and radiology during the 1980s and 1990s. Effective dose has provided a valuable tool that is now used in the establishment of guidelines for patient referral and justification of procedures, choice of appropriate imaging techniques, and providing dose data on potential exposure of volunteers for research studies, all of which require the benefits from the procedure to be weighed against the risks. However, the approximations made in the derivation of effective dose are often forgotten, and the uncertainties in calculations of risks are discussed. An ICRP report on protection dose quantities has been prepared that provides more information on the application of effective dose, and concludes that effective dose can be used as an approximate measure of possible risk. A discussion of the way in which it should be used is given here, with applications for which it is considered suitable. Approaches to the evaluation of risk and methods for conveying information on risk are also discussed.

## 1. INTRODUCTION

The dose quantities for medical procedures using ionising radiations that are measured cannot readily be used to compare exposures in relation to risk. Radiation exposures are seldom uniform over the whole body and usually involve irradiation of several organs and tissues; this has a significant effect on the relative risks. However, the radiations used for diagnostic and interventional procedures are always x rays, gamma rays, beta particles, or positrons. Therefore, the differences in biological effectiveness of the radiations in damaging tissue are relatively small and have less influence on the risk. The concept of combining doses to individual organs weighted according to their sensitivity to induction of stochastic effects in order to derive an effective dose linked to risk was first proposed in 1975 (Jacobi, 1975). The International Commission on Radiological Protection (ICRP) developed this principle further, and recommended derivation of a dose-equivalent limit based on the total risk to all tissues irradiated, linked to stochastic effects derived from results of epidemiological studies. The approach stems from the principle that the risk associated with the dose quantity should be equal to that from a similar uniform dose to the whole body. This was achieved by summing doses to individual tissues, each modified by a tissue weighting factor based on an assessment of the risk from stochastic effects, namely cancer and genetic effects (ICRP, 1977). A remainder was included consisting of an average dose for other tissues that were potentially at risk from cancer induction. The initial dose quantity was called the ‘effective dose equivalent’ and was applied in the evaluation of doses received by radiation workers and the public. The primary organs at risk that were included were the gonads for genetic effects, and the breast, lung, and bone marrow for cancer, with lower weighting for the thyroid and bone surfaces relating to malignancy. It was used to provide a method for judging the acceptability of the level of risk in radiation work by allowing comparisons of the risk from radiation exposure with the risks for other occupations, as well as planning of operations and optimisation of procedures to keep dose levels to radiation workers and the public at acceptable levels. The cancer risk data used in derivation of the tissue weighting factors are largely from the Life Span Study of the Japanese survivors from the atomic bombs detonated in 1945. The lifetime risks of developing cancer from exposure of different organs compared with dose data, which appear linear between doses of <100 mGy to several Gy, are extrapolated down to low doses (ICRP, 2005; Shore et al., 2018). This linear no-threshold (LNT) model is used to calculate the probability of radiation-induced cancer for organs and tissues for which there are sufficient data (ICRP, 2007).

ICRP renamed the quantity ‘effective dose’ when the fundamental recommendations on radiological protection were updated (ICRP, 1991). Changes were made in the organs/tissues included in the effective dose and the tissue weighting factors because of growing evidence of links between cancers in other tissues and radiation exposure identified through the Life Span Study. Further modifications in the formulation were made in the last set of fundamental recommendations based on changes in analyses of the epidemiological data and the calculation of radiation detriment (ICRP, 2007). The weighting factors are rounded to facilitate calculation in order to provide a radiation protection dose quantity that is easy to apply in practice. Thus, effective dose is a protection quantity designed for easy application, rather than a scientific quantity that can be measured, and is acknowledged to be an approximation with inherent uncertainties (Martin, 2007; McCollough et al., 2010).

## 2. DOSE QUANTITIES USED IN MEDICAL APPLICATIONS

### 2.1. Measurable dose quantities

Radiation is used in a wide range of applications in medical diagnosis and therapy. For diagnostic and interventional x-ray applications, radiation doses received by patients are recorded in terms of quantities that can be measured and are generally displayed on equipment consoles. For radiography and fluoroscopy, they take the form of entrance surface air kerma, that relates to dose to the skin surface, and kerma-area product (KAP; P_KA_), that gives a measure of all radiation incident on the patient. For computed tomography (CT), they take the form of the CT dose index that is associated with doses to the tissues within the section of the body being imaged, and the dose length product (DLP) that gives a measure of dose from a whole procedure. These measured quantities can be recorded and applied readily for assessment of dose levels, and are used for collection of data in patient dose surveys, comparisons of doses for examinations at different healthcare facilities, optimising procedures, and setting diagnostic reference levels (Martin, 2011a; ICRP, 2017). In fact, they are useful for most applications where a measure of dose is required, such as for recording patient dose information in medical reports, as required by European member states (EU, 2014) and for joint common accreditation in the USA, and for tracking doses to individual patients accumulated over time (Rehani et al., 2014). The activities of radionuclides, together with the type of radiopharmaceutical administered to each patient, fulfil the same roles in nuclear medicine.

### 2.2. The need for and evolution of effective dose in medicine

In medicine, imaging examinations using ionising radiations are performed on different parts of the body to aid diagnosis and treatment of a wide range of diseases. Judgements have to be made about examinations relating to the level of risk, but radiation quantities that can be measured often give little indication of potential risk. Comparison of KAP values for the chest and abdomen do not have much relevance, nor do comparisons of KAP and DLP for the same body region, or the measured dose for an x-ray procedure with the amount of radioactivity administered for a nuclear medicine examination. In all these cases, the distributions of radiation doses to organs and tissues within the body will be very different. There is a need for a dose quantity that supplies some information on risk to inform decisions about the appropriateness of radiation exposures used for diagnosis and management of treatments for large numbers of patients.

Effective dose was designed as a protection quantity to enable decisions to be made about potential exposures of workers and the public, and to set dose limits, constraints, and reference levels. However, ICRP acknowledged that it could provide a useful measure of doses to nuclear medicine patients in whom radionuclides accumulated in various organs around the body, and that its use could facilitate comparisons between different types of medical radiological investigation (ICRP, 1987). Since that time, ICRP Committees 2 and 3 have collaborated to derive coefficients to enable absorbed doses to organs and tissues, and assessments of effective doses received by nuclear medicine patients to be quantified in order to fill this gap. The reports use biokinetic models developed from available data within a generic framework to evaluate the activities of different radionuclides that are likely to accumulate in different organs (e.g. ICRP, 1988, 2015). Radionuclide distributions and transit times through different organs are evaluated and activity–time curves generated. These are used, together with mathematical models of the anatomy for a reference person, to obtain absorbed doses for all the organs and tissues within the body from the accumulated activity in the ‘source’ organs. Coefficients have since been published to enable calculation of organ and effective doses for diagnostic x-ray procedures by a number of organisations, and these can be applied to the entrance surface air kerma or KAP for radiography and fluoroscopy (Hart et al., 1994; Ranniko et al., 1997; Kramer et al., 2004), or the DLP for CT (Ding et al., 2015; Shrimpton et al., 2016). Values of effective dose calculated with these coefficients can be used to compare doses from a wide range of medical procedures that expose different regions of the body, and these have been instrumental in raising awareness of dose levels from diagnostic imaging procedures among medical physicists, clinicians, and radiographers.

### 2.3. The application of effective dose to medical patients

Effective dose is now used in training medical professionals in radiological protection, and can provide a broad understanding of possible risks associated with radiation exposures. It has provided a universal dose quantity that can be used as a reference against which improvement in radiological protection in medical practice can be judged, and gives an indication of radiation dose relating to possible risks to health that can be understood by clinicians and non-specialists in radiological protection. The details of how, and for what purposes, effective dose is applied vary across the world, but include decisions made as part of the process for justifying imaging exposures for individual patients and optimising protection through selection of the most appropriate technique. Generic values of effective dose calculated for common procedures provide a straightforward tool that can be used for making these everyday decisions.

However, the application of effective dose to medical procedures is rather different from occupational and public applications, in which the requirement is for a measure relating to risk that can be used in the optimisation of protection below constraints or reference levels. With medical x rays, the exposure is planned, limits on the region of the body exposed are defined, and simulations are used to evaluate doses to individual organs, although these are in reference anatomical phantoms rather than the patient. Thus, more is known about the dose distributions from medical exposures than those to workers. This creates the impression that the doses to patients are known with much greater certainty than they actually are, and has led to users losing sight of the many approximations employed in the derivation of effective dose (Martin, 2007; McCollough et al., 2010). In the use of effective dose for evaluation of occupational and public doses, there had been little need to consider these uncertainties.

## 3. UNCERTAINTIES IN CALCULATIONS OF EFFECTIVE DOSE AND RISK

### 3.1. Approximations made in the derivation of effective dose and its use in medicine

As effective dose can express dose in terms related to relative risk from exposures of different parts of the body, it is admirably suited for application to medical exposures. However, approximations involved in the derivation and uncertainties in the calculation need to be taken into account in its application to assessment of doses to patients.

#### 3.1.1. Age and sex

The risk estimates to which effective dose relate have been derived for populations of all ages, so while medical exposures may relate to individuals, effective dose applies to a sex-averaged reference person exposed in the same way (ICRP, 2007).

#### 3.1.2. Tissue weighting factors

As effective dose is a practical operational tool, the most important requirement is for it to be simple to calculate and use. Tissue weighting factors are rounded approximations related to the risks that stem from epidemiological data that are judged to be acceptable for deriving a radiation protection dose quantity. Differences from the risks calculated from epidemiological data are an approximation that give another source of inaccuracy.

#### 3.1.3. Dose measurements

Values for effective dose are computed from the results of practical measurements of dose quantities in which there are uncertainties. For example, for x-ray exposures, there will be uncertainties not only in the tissue dose measurement itself, but also in the extent of the region of the body exposed that relates to the size of the x-ray field.

#### 3.1.4. Computations

The derivation of effective dose requires values for doses to all of the organs exposed to be computed, and this is done via Monte Carlo simulations. There are significant uncertainties in these calculations that combine with those in the boundaries of the radiation fields in radiology (Martin, 2007). For nuclear medicine examinations, uncertainties in the radionuclide dose transit time curves include factors such as the time that radioactivity remains in the bladder, which is dependent on the patient’s actions. They also depend on patient anatomy, which determines the proximity of organs for which absorbed dose is being assessed to those in which radioactivity accumulates (Martin, 2011b).

The net result is that there may be an uncertainty of ±40% in values derived for effective dose as a relative indicator of risk to a reference person when applied to medical imaging procedures in general (Martin, 2007). For some diagnostic nuclear medicine investigations where the dose to the target organ, or to the bladder and colon irradiated during the excretion process, represents a significant proportion of the total dose, the uncertainty may be ±50% (Martin, 2011b). When this is considered in terms of radiation exposure and risk in general, the magnitude of the uncertainty is not unreasonable, and effective dose provides a useful comparator for making overall judgements about the relative risks from different types of medical procedure and making comparisons with doses from other sources. However, because of the uncertainties, effective dose should only be quoted to one significant figure for values less than 1 mSv, and two significant figures for values above 1 mSv.

Effective dose is the only relatively simple way in which a dose with some link to risk can be expressed, but users must acknowledge its approximate nature, use it as a guide in making decisions and steering practice, and recognise that it has large uncertainty and applies to a reference person rather than an individual. Comparisons of effective doses for medical procedures with everyday exposures from natural background radiation and from cosmic rays during a plane flight, to which people can relate, is sometimes helpful. These comparisons can be particularly useful in discussions with patients who have little or no knowledge about radiation, and may have an unrealistic fear of the potential harm from an exposure.

When using effective dose, it should be borne in mind that the potential risk for patients from medical exposures is generally lower than that for a reference population due to their higher average age and the reduced life expectancy due to disease (Loose et al., 2009). However, risks for paediatric patients are generally higher and this potential increased sensitivity should be recognised (ICRP, 2013).

In medical examinations where only one organ is exposed, estimates of the dose to that organ or tissue should be used instead of effective dose. Examples are radiological imaging of anatomic areas outside the trunk, such as the breast in mammography or the brain in head CT. This also applies to radioiodine uptake by the thyroid, quoted in terms of absorbed dose to the thyroid, and gonad dose where this makes up the majority of the dose received.

### 3.2. Use of effective dose in conveying radiation risk

Risks of cancer incidence relating to effective dose are quoted in the fundamental recommendations of ICRP (ICRP, 2007). These are helpful in providing a calibration of effective dose in terms of risk. In a forthcoming ICRP publication on use of the protection quantities, it is concluded that effective dose can be used as an approximate measure of possible risk. This wording was chosen to emphasise the uncertainties inherent in the estimation of risk, and to acknowledge that the doses under consideration are, in many cases, below the levels at which direct epidemiological observations of excess cases of cancer are available.

#### 3.2.1. LNT model

Effective dose employs the LNT model, as this is considered to be the best approach to quantifying the risk–dose relationship on the basis of current knowledge (ICRP, 2005; NCRP, 2018; Shore et al., 2018). By assuming that the lifetime risk of cancer is directly proportional to the dose, doses from all radiation exposures can be summed. This means that small radiation doses well below the level at which any effect can be demonstrated are taken into account and considered to be potentially harmful. It is not possible to prove a definitive form for the link between exposure and cancer at these dose levels, as this would require study of populations of tens of millions of individuals whose exposures were known, together with matched control groups. The uncertainty in the LNT model applies to any calculation of risk at low doses, whether calculated from effective dose or doses to individual organs.

#### 3.2.2. Adjustments for exposed populations

Risks of cancer originate from epidemiological studies, predominantly of the Japanese survivors of the atomic bombs detonated over Hiroshima and Nagasaki. Recent cancer risk vs dose models have been constructed from mortality data for leukaemia and cancer incidence data for solid tumours (BEIR, 2006; ICRP, 2007; Berrington de González et al., 2012). Two approaches are used to obtain projections of lifetime risk. The first, called the ‘excess absolute risk’ (EAR) or ‘additive risk model’, assumes that the excess absolute risk is proportional to the dose to the tissue. The second, called the ‘excess relative risk’ (ERR) or ‘multiplicative model’, includes an adjustment linked to the relative rates of cancer incidence in the target population and the unexposed study population (ICRP, 2007). The target population used by ICRP aims to provide global average values, and bases its assessments on a composite population comprising four Asian populations, two European populations, and a US population. Risks for the breast are based on the EAR model, risks for the thyroid and skin are based on the ERR model, risks for the lung are based on an ERR:EAR weighting of 0.3:0.7, and risks for other organs are based on a 0.5:0.5 ratio. Decisions about the weighting stem from the expert opinion of members of the committee formulating the values. Risks per unit organ dose published in BEIR (2006) and Berrington de González et al. (2012) differ from those in ICRP (2007) as they use a US population with slightly different factors (Martin, 2019).

#### 3.2.3. Dose and dose rate effectiveness factor

Radiobiological experimental investigations have tended to show that risks are reduced for fractionated or protracted exposures, suggesting that high-dose, acute exposures may overestimate the risk of cancer induction. Therefore, in ICRP (2007), the risk estimate is divided by 2.0, but this is again an approximation that stems from earlier methods and views of Commission members. The value used in BEIR (2006) and Berrington de González et al. (2012) for risk calculations is 1.5.

#### 3.2.4. Other uncertainties

There are other sources of uncertainty in any risk estimates. For example, there will be interactions between radiation exposure and other cancer risk factors, notably smoking history in the case of lung cancer, and reproductive history in the case of female breast cancer. Another example is the assumption inherent in the application of a single radiation weighting factor of 1 to describe the relative biological effectiveness for all photon radiations in the 30–200-keV range (Heyes et al., 2009) and beta-particle radiations.

There has been a desire to quote risks from radiation exposure in numerical terms in many countries, and effective dose has been used to calculate a figure for the excess lifetime risk of cancer. However, even for the ICRP reference person, actual risks might be three times higher or lower than the estimate, but the uncertainty could be much greater given the lack of definitive proof for the LNT model at low doses. The use of medical radiation has been increasing rapidly over the last 20 years, and there is a need to try to reduce numbers of unnecessary exposures. In promotion of this message, claims have been made quoting large numbers of additional cancers that could result from this increase (Brenner and Hall, 2007; Berrington de González et al., 2009). These numbers are derived using the BEIR (2006) model, but with little account taken of the uncertainties in epidemiological data, the extrapolation to low doses, or the reduced life expectancy of patients because of their illnesses. Such numerical assessments can give a false impression of accuracy, and should be appropriately caveated with consideration of uncertainties and background rates. The use of general terms linked to possible levels of cancer risk, as shown in Table 1, avoids the impression of precision in risk estimates. These terms are considered to be reasonable indications of the risk from cancer induction for those aged between approximately 20 years and 60 years. When using these terms in discussions about patients, the influence of their disease, condition, and age on life expectancy should be taken into account.

**Table 1.** Dose ranges and terminology for describing the excess lifetime risks of cancer incidence from different medical diagnostic procedures for adult patients of average age (30–39 years).

Effective doses (mSv)	Risk of cancer inferred from LNT model^*^	Proposed term for dose level	Examples of medical radiation procedures within different dose categories
<0.1	<10^−5^	Negligible	Radiographs of chest, femur, shoulder limbs, neck, and teeth
0.1–1	10^−5^–10^−4^	Minimal or extremely low	Radiographs of spine and trunk, and ^99m^Tc lung ventilation and renal imaging
1–10	10^−4^–10^−3^	Very low	CT scans of head and body, cardiac angiography, and a variety of nuclear medicine examinations
10–100	10^−3^–10^−2^	Low	Multiple CT scans of trunk with contrast and higher dose interventional procedures
100 s	>10^−2^ based on epidemiology	Moderate	Multiple procedures and follow-up studies

LNT, linear no-threshold; CT, computed tomography.

* Risk bands are lifetime detriment adjusted incidence to nearest order of magnitude.

### 3.3. Application of risks to individual patients

The risk estimates used in the derivation of effective dose have been age- and sex-averaged. Some of the differences between risks for an individual and those for the ICRP reference person can be taken into account if required. The differences are listed below.

#### 3.3.1. Age

Overall lifetime risks of cancer from radiation exposure decline with age, with risks for exposure of children aged 0–10 years being approximately double those for exposure in middle-aged adults (30–50 years), and those for the over 60 s being approximately half. The greater radiosensitivity of tissues in children contribute to their higher risk, but variations with age at exposure primarily reflect differences in the remaining lifetime after exposure. There are substantial differences between cancer types, with risks of lung cancer induction increasing in middle age, and risks of thyroid and female breast being high for the young and falling to a low level by 30–40 years (ICRP, 2007).

#### 3.3.2. Sex

Lifetime cancer risks differ for the two sexes, with the significant risks of breast cancer applying virtually exclusively to females. In addition, risks of thyroid cancer are four to five times greater in females, and risks of lung cancer are almost double. For cancers such as colon and leukaemia, the risk in males is 40–50% higher.

#### 3.3.3. Health status

Patients undergo examinations to investigate disease, and in many cases, the medical risk from their condition is likely to reduce their life expectancy and therefore the risk of radiation-induced cancer (Loose et al., 2009).

#### 3.3.4. Genetic factors

There are known to be differences in genetic susceptibility to cancer, with certain sections of the population likely to be more susceptible to cancer induction by radiation.

Epidemiological data have been used to determine risks from exposure of individual organs and tissues within the body (BEIR, 2006; ICRP, 2007), so if a more accurate assessment of risk is deemed necessary, this can be calculated using the risk coefficients for each organ and tissue separately, based on the age and sex of the exposed individual. Brenner (2008, 2012) proposed the use of the term ‘effective risk’ to describe an approach to the summation of risks estimated in this way. However, while this approach uses the available data on age- and sex-specificity of the different cancer types, it does not take account of the large uncertainties described in Section 3.2.

## 4. APPLICATIONS OF EFFECTIVE DOSE IN MEDICINE

In its forthcoming publication on protection dose quantities, ICRP has set out the purposes for which use of effective dose is recommended in medicine, and these are given below.

### 4.1. Referral guidelines and justification of procedures

Effective dose provides information on relative magnitudes of doses from different types of examination that can be used in referral guidelines and in justification of techniques at national level. In addition, it can be used by clinicians in making decisions as part of the justification of procedures for individual patients. Effective dose provides sufficient information to allow clinicians to weigh the benefit from the diagnostic information needed for management of the patient’s disease against the potential risk from radiation exposure, taking account of the sex, age, medical risk from their condition, and life expectancy (Loose et al., 2009).

### 4.2. Choice of imaging technique

Effective dose enables doses from procedures in which the dose distributions are different to be compared (e.g. x ray and nuclear medicine). Decisions about which technique to use will be based primarily on the type of information each will provide for the potential benefit to the patient, but the relative effective dose is a secondary factor that can be taken into account when appropriate.

### 4.3. Optimisation of technique

In general, effective dose is not the best quantity for making comparisons between doses for similar techniques for which there are measurable quantities such as KAP or DLP. However, if the dose distribution within the body changes, because of radiographic projection, tube potential, or addition of a filter, effective dose may be useful for evaluating changes in exposure of the different organs and tissues.

### 4.4. Doses to research volunteers

Before a research proposal is approved, the possible detriment for the individuals involved should be evaluated and recorded (ICRP, 1992). Effective doses from the various radiation procedures that are to be performed can be summed to give an indication of the possible overall radiation-related health detriments that may accrue to the volunteers. Effective dose is particularly useful because the procedures performed may involve different dose distributions within the body, but it should be recognised that it is estimated for a reference person, so when considering the potential radiation-related risks, the age, sex, and health status of the volunteers should be taken into account.

### 4.5. Reporting of unintended exposures

Effective dose can provide enough information for assessments of unintended exposures and overexposures of patients in diagnostic procedures due to procedural errors or equipment faults. It can be assessed during incident investigations and included in reports (Martin et al., 2017). For more substantial exposures that may approach or exceed 100 mSv, estimates of risk using the best scientific data will be appropriate.

### 4.6. Efficacy of imaging for health screening or non-medical applications

Effective dose can be used in the evaluation of health screening procedures that involve exposure of many organs within the trunk.

### 4.7. Doses to carers

Medical exposures are considered to include exposures incurred knowingly and willingly by individuals helping in the support and comfort of patients undergoing diagnosis or treatment. This application is more akin to that in occupational exposure, and methods for the prediction of values for effective dose are similar. A typical example where this might be required is the exposure of family members from a patient discharged after thyroid treatment with unsealed ^131^I. The effective doses that might be received by the individuals involved and the acceptability will be determined by the individual circumstances (ICRP, 2007).

### 4.8. Education and training of clinicians and other healthcare professionals

It is often difficult for clinicians who refer patients and perform medical procedures involving radiation to take potential risks into account when requesting or justifying patient diagnostic or interventional exposures (ICRP, 2009; Loose et al., 2009; Zanzonica and Stabin, 2014). Effective dose provides a single value which can be used to compare different exposure scenarios, and a knowledge of typical effective doses from common procedures should be included in the education and training of medical practitioners. Effective dose is an appropriate quantity for straightforward communication when explaining possible risks to patients, and allows comparisons of the possible health risks of an exposure with risks from other exposure scenarios.

### 4.9. Use of collective effective dose for medical exposures

Effective dose has been used in evaluating the level of exposure in different countries (UNSCEAR, 2008). The use of collective effective dose in this way has been used for deriving average population dose per caput from medical exposures. It has contributed to the raising of awareness of doses from medical procedures in the USA (NCRP, 2009, 2019) and UK (Wall et al., 2011), and optimisation efforts following on from these surveys have led to significant reductions in doses from medical procedures. However, extending the use of collective effective dose to predict health effects should be treated with caution.

## 5. CONCLUSIONS

Effective dose in medicine provides a tool that can aid judgements that have to be made about diagnostic examinations and patient management relating to the level of risk. Values of effective dose can be derived from measurable quantities and comparisons made between medical procedures using different imaging modalities or exposing different regions of the body. Effective dose has proved to be a valuable tool in medicine, providing a single dose quantity for communication with clinicians and patients. Doctors who refer patients or perform medical procedures involving radiation may have little understanding of the potential health detriment from radiation exposure, and a knowledge of typical effective dose values for common medical procedures is used in training medical professionals and informing judgements on relative radiation dose levels. Such information is then used in making everyday decisions; for example, as part of the referral and justification process for imaging exposures for individual patients, and in the selection of appropriate imaging techniques.

A forthcoming ICRP publication discusses the use of protection quantities, and concludes that effective dose can be used as an approximate indicator of possible risk. There are substantial uncertainties in the estimation of risk at low doses, recognising that the doses under consideration are likely to be below the levels at which direct epidemiological observations of excess cases of cancer are available. However, the most straightforward interpretation of the available scientific evidence for the purposes of radiological protection is that a nominal lifetime fatal cancer risk estimate of approximately 10^-4^–10^-5^ per Sv applies at low doses or low dose rates. The evidence also shows differences in risk between males and females, and particularly with age at irradiation. Such differences can be taken into account when considering risks to individuals. It is emphasised that situations that require best estimates of risk should be evaluated using the best scientific data – including organ/tissue absorbed doses; relative biological effectiveness estimates; and age-, sex- and population-specific risk estimates – with consideration of uncertainties.

The use of effective dose has helped to raise awareness of dose levels from diagnostic imaging procedures among healthcare staff. However, users often forget the approximations made in the derivation of effective dose, and overstate its accuracy. Effective dose is only accurate to perhaps ±40% as a relative indicator for a reference person; as such, it should not be stated to more than two significant figures. Use of effective dose to predict the risk of cancer induction from a low-dose radiation imaging procedure introduces much greater uncertainties, so descriptive terms are recommended for conveying risk which reflect uncertainties in risk predictions. These terms are sufficient in many cases because the risks from most medical diagnostic exposures are small. If it is considered necessary to calculate a more accurate assessment of risk, this should be based on doses to all of the exposed organs and risk coefficients used for a person of the same age and sex, with appropriate consideration of uncertainties.
